# Delay-Embedded Neural Reconstruction for Indirect Sensing in Electrical and Micromechanical Oscillating Systems †

**DOI:** 10.3390/s26144504

**Published:** 2026-07-15

**Authors:** Francesco Grimaldi, Christian Geminiani, Andrea Tilli

**Affiliations:** Department of Electrical, Electronic and Information Engineering (DEI), Alma Mater Studiorum, University of Bologna (UniBo), 40136 Bologna, Italy; christian.geminiani2@unibo.it (C.G.); andrea.tilli@unibo.it (A.T.)

**Keywords:** takens embedding, oscillating sensors, neural reconstruction, MEMS gyroscope, indirect sensing

## Abstract

This paper addresses indirect sensing in resonant, oscillating, and periodically forced sensors, where the physical measurand is not directly available as a static output but is encoded in the dynamic response of the device. The sensor and its excitation are described as a single autonomous system, in which the excitation phase and the slowly varying measurand define a compact state representation after the decay of transients. Within this setting, delayed samples of the available output define an observation map that can be inverted, under suitable smoothness and observability conditions, to reconstruct the measurand in a deadbeat-like fashion. Compared with a preliminary conference study based on a simplified scalar-output RLC benchmark, the present work extends the formulation to vector-valued outputs, introduces a local conditioning indicator based on the Jacobian matrix, and focuses on a micromechanical sensing case with nonlinear electromechanical transduction. The inverse observation map is approximated by a feedforward neural network trained on synthetic data generated from the autonomous model. The methodology is applied to a vibratory MEMS gyroscope, where the signed angular rate is reconstructed from a delayed-output sequence combining the nonlinear capacitive current readout and the known AC drive reference. The augmented output is introduced to overcome the lack of observability affecting the raw current signal over signed angular-rate ranges. Numerical results show accurate reconstruction in ideal conditions and provide a preliminary robustness assessment under additive output noise.

## 1. Introduction

Resonant, oscillating, and periodically forced sensors are widely used when a physical quantity must be inferred from the dynamic response of a mechanical, electrical, or electromagnetic structure. In these devices, the measurand is not directly available as a static output. Instead, it modifies a dynamic signature of the sensor, such as a resonance frequency, a phase shift, a quality factor, an oscillation amplitude, or the harmonic content of a forced response. This makes oscillating sensors attractive for high-sensitivity measurements, digital processing, and wireless or remote interrogation, but also makes the reconstruction problem intrinsically nonlinear and strongly dependent on the adopted transduction mechanism.

Examples range from self-oscillating sensors, such as atomic and nuclear-magnetic-resonance magnetometers, where the target quantity is encoded in the natural oscillation frequency of the system [1,2], to externally forced resonant sensors, where the excitation is imposed and the measurand appears in the steady-state response: quartz and SAW resonator sensors [3], resonant MEMS microsensors [4], MEMS vibratory gyroscopes [5], and wireless passive LC or RFID-like sensors for harsh environments [6]. Despite their different physical implementations, these devices share a common feature: after transients have decayed, the useful information is carried by a low-dimensional oscillatory response whose shape depends on the unknown physical parameter.

Conventional reconstruction strategies usually rely on sensor-specific analytical models and dedicated signal-processing pipelines; representative approaches include coherent demodulation-based methods [7], Kalman-filter-based observers [8], and direct model inversion. Demodulation techniques are widely adopted because of their simplicity, robustness, and limited dependence on an accurate sensor model; however, the low-pass filtering stage required for coherent demodulation inherently introduces latency and limits the achievable bandwidth, and these schemes are generally tailored to the estimation of a single quantity, without naturally providing access to additional dynamic states of the resonator. Model-based observers, such as Kalman and Extended Kalman Filters, exploit a state-space representation of the sensor dynamics to reconstruct multiple physical quantities simultaneously, but their performance strongly depends on the accuracy of the underlying model and on the appropriate tuning of the process and measurement noise covariance matrices. In addition, since the filter recursion must be executed in real time, the complexity of the sensor model that can be embedded in the observer is bounded by the available online computational budget. Direct model inversion reconstructs the quantity of interest directly from the sensor dynamics, but is typically highly sensitive to measurement noise and model inaccuracies.

The objective of the present work is to provide a common reconstruction workflow, based on delay-coordinate embedding and learned inverse mapping, that transfers across heterogeneous resonant sensing technologies while remaining independent of the specific transduction principle. Within this workflow, the learned inverse mapping can be configured to estimate several physical quantities simultaneously from the same embedded representation, without designing a dedicated pipeline for each target variable, and, unlike Kalman-based approaches, it does not require tuning of the process and measurement noise covariance matrices, a task that can be quite challenging in practice. Moreover, since the inverse map is learned offline, the model used to generate the training data can be made arbitrarily complex and accurate without affecting the online computational cost, which reduces to a single forward evaluation of the trained network. These features motivate the use of Takens’s delay-embedding framework [9,10], in which the sensor and its excitation are treated as a single autonomous dynamical system, and the measurand is reconstructed from a finite sequence of delayed output samples.

The sensor excitation is embedded into the model, while the measurand is regarded as a slowly varying state over the considered observation window. Under suitable smoothness and observability assumptions [11], delayed samples of the available output define an observation map from the compact sensor state to a finite-dimensional output manifold. If this map is injective and sufficiently well conditioned, the current state can be recovered in a deadbeat-like fashion by approximating its inverse [12,13].

Since the analytical inverse of the delay map is generally unavailable, the inverse reconstruction is learned from synthetic data generated from the autonomous sensor model. A feedforward neural network is used as a practical function approximator, while a local observability indicator, the minimum eigenvalue of $J^{⊤}J$, is introduced to assess the conditioning of the delay-output map through a compact numerical measure of local rank deficiency or poor conditioning. This analysis not only represents a necessary condition test but can also provide support in the selection of excitation frequencies, sampling times, operating ranges, and design of sensor parameters.

While a preliminary version of this work was presented in a conference paper focused on a simplified temperature-dependent RLC benchmark [14], the present article builds on the same delay-embedding idea and extends it toward a more realistic micromechanical sensing problem: the formulation is expanded from scalar outputs to vector-valued outputs, allowing the reconstruction algorithm to exploit additional measured channels or known reference signals; the observability analysis is reformulated through a local conditioning indicator based on the minimum eigenvalue of $J^{⊤}J$; and the application study is focused on a vibratory MEMS gyroscope in which the signed angular rate is reconstructed from an augmented delayed-output sequence.

The MEMS gyroscope benchmark is selected because it introduces a physically meaningful observability issue that does not arise in the preliminary scalar-output RLC setting: the signed angular rate is encoded in the oscillatory sense response generated by Coriolis coupling, while the capacitive current readout alone may lead to sign ambiguities over the considered state domain. This makes the benchmark suitable for showing the role of output augmentation, in which the nonlinear capacitive readout is combined with the known AC drive reference, providing phase information and improving the informativeness of the delay-output map.

The remainder of the paper is organized as follows. Section 2 introduces unified autonomous models for self-oscillating and externally forced sensors. Section 3 presents the delay-embedding reconstruction strategy, its extension to vector-valued outputs, the local observability indicator, and the neural inverse approximation. Section 4 applies the methodology to a MEMS vibratory gyroscope for signed angular-rate reconstruction, including ideal reconstruction and a preliminary robustness test under output noise. Finally, Section 5 summarizes the main results and discusses future developments.

## 2. Typical Resonator Models

As discussed in the Introduction, oscillating sensors can be broadly divided into two categories. Self-oscillating active sensors exhibit a natural limit-cycle oscillation, whose frequency or orbit depends on the target variable. Forced-oscillating sensors, instead, are driven by an external periodic input and converge to an attractive oscillatory steady state associated with the forcing signal. In both cases, let $p\in R^{n_{p}}$ denote the target variable, or vector of target variables, $w={\left[w_{1}\;w_{2}\right]}^{⊤}\in R^{2}$ the oscillator coordinates, and $y\left(t\right)\in R^{q}$ the available output vector. The case $n_{p}=1$ corresponds to the reconstruction of a single physical quantity, such as the angular rate in the MEMS gyroscope benchmark considered in this work, while q>1 allows the reconstruction algorithm to exploit multiple measured channels or known reference signals available from the excitation electronics.

Since the target variable is assumed to evolve slowly compared with the intrinsic sensor dynamics, i.e., there is a clear time-scale separation, it is treated as a constant parameter over the finite observation window used for reconstruction. When the time variation in *p* is not negligible over this window, the induced non-stationarity can be addressed through overembedding techniques, where the delay-coordinate space is enlarged so as to implicitly account for the additional parameter degrees of freedom [15].

### 2.1. Self-Oscillating Active Sensor Model

A self-oscillating sensor can be represented by

(1) $$\dot{w}\left(t\right)=S\left(p\right)w\left(t\right)\;S\left(p\right)=\left[\begin{array}{cc} 0 & -\omega (p)\\ \omega (p) & 0 \end{array}\right]\;{∥w\left(0\right)∥}^{2}=1\;y=h\left(w,p\right)$$

Here and in the following, the output map *h* is allowed to be vector-valued, namely $h\left(\cdot \right)\in R^{q}$. The initial-condition constraint forces the trajectory of *w* to remain on the unit circle, so that the oscillation can be equivalently described by a single phase variable.

### 2.2. Forced-Oscillating Sensor Model

For a forced-oscillating sensor, the excitation is generated by an autonomous oscillator and injected into the sensor through a known input map:

(2) $$\dot{w}\left(t\right)=Sw\left(t\right)\;S=\left[\begin{array}{cc} 0 & -\omega \\ \omega & 0 \end{array}\right]\;{∥w\left(0\right)∥}^{2}=1\;u=u\left(w\right)$$

The forced sensor dynamics are described by

(3) $$\dot{x}\left(t\right)=f\left(x,p,u\right)\;y=h\left(x,p\right)$$

where *x* denotes the internal sensor state. In the forced case considered here, the input is not an arbitrary unknown signal, but an imposed periodic excitation generated by the exosystem (2). The role of *w* is therefore to make the forced sensor autonomous and to keep track of the excitation phase. The corresponding augmented state is the following:

(4) $$\chi ={\left[\begin{array}{ccc} x^{⊤} & p^{⊤} & w^{⊤} \end{array}\right]}^{⊤}$$

The reduction of the forced dynamics to a compact autonomous representation relies on an attracting steady-state response. Following Stark’s invariant-graph framework for forced systems [16], we assume that, for each fixed value of the slowly varying parameter *p* and for each prescribed forcing trajectory u(w), the forced subsystem is contractive with respect to its initial state. Thus, the response asymptotically loses memory of its initial internal condition and becomes uniquely determined by the forcing phase and by the parameter value x=φ(w,p).

Under this assumption, the asymptotic forced response is represented by an attracting invariant graph, describing the steady-state response reached after transient decay, where the dependence on *p* is the target variable to be reconstructed: once the transients have vanished, the internal sensor state is constrained by the imposed excitation phase and by the slowly varying measurand. The convergence toward the graph is assumed to be sufficiently fast with respect to the forcing period 2π/ω and with respect to the time scale over which *p* varies. Hence, over the finite delay window used for reconstruction, *p* can be regarded as constant. The forced sensor can then be restricted to its steady-state invariant graph, leading to the reduced representation.

(5) $$\dot{w}\left(t\right)=Sw\left(t\right)\;{∥w\left(0\right)∥}^{2}=1\;x\left(t\right)=\varphi \left(w,p\right)\;y=h\left(\varphi \left(w,p\right),p\right)=\tilde{h}\left(w,p\right)$$

The contractivity assumption restores the deterministic one-to-one dependence of the observations *y* on the forcing state *w* and *p*. As all trajectories collapse onto a low-dimensional manifold governed by the forcing dynamics and the target variable, Takens’s embedding theorem can be applied directly to reconstruct the target parameter [9,10].

### 2.3. Unified Oscillating Sensor Model

Under the frozen-parameter approximation introduced above, the measurand can be regarded as constant over the delay-embedding window used for reconstruction. Therefore, after transient decay in the forced case, both self-oscillating and forced-oscillating sensors can be treated within the following reduced autonomous description:

(6) $$\dot{p}\left(t\right)=0\;\dot{w}\left(t\right)=S\left(p\right)w\left(t\right)\;{∥w\left(0\right)∥}^{2}=1\;y=h\left(w,p\right)$$

(7) $$S\left(p\right)=\left[\begin{array}{cc} 0 & -\omega (p)\\ \omega (p) & 0 \end{array}\right]$$

Here $\dot{p}\left(t\right)=0$ is again a local, finite-window assumption rather than global constancy of the measurand. In self-oscillating sensors ω(p) may depend on the measurand, whereas in forced-oscillating sensors ω is imposed by the external excitation and is independent of *p*. The sensor-excitation system is assumed to satisfy the structural conditions required by the method, namely sufficient smoothness of the dynamics and output map, local state distinguishability from output trajectories, and the time-scale separation stated above, all ensured at the design stage.

## 3. Nonlinear Deadbeat-like Reconstruction via Neural-Network Approximation

Under the invariant-graph reduction introduced in Section 2, the relevant state space for forced sensors is the compact manifold parameterized by the excitation phase and by the slowly varying measurand: the delay-embedding framework is therefore applied to the reduced state (ϑ,p), with dimension $n=1+n_{p}$, after the exponentially decaying internal transients have been removed by the fiber-contraction property.

In the remainder of this section, and in the MEMS benchmark considered in Section 4, the formulation is specialized to the scalar-measurand case $n_{p}=1$. The main difficulty lies in the output function h(w,p), which may be highly nonlinear and sensor-dependent. Since the oscillatory state $w={\left[w_{1}\left(t\right)\;w_{2}\left(t\right)\right]}^{⊤}$ evolves on the unit circle, it can be equivalently parameterized by a single phase-angle variable ϑ(t); the redundant two-dimensional oscillator description is therefore replaced by the compact state x=(ϑ,p), where *p* is the slowly varying scalar target variable, so that the effective autonomous model has dimension n=2 on the invariant oscillatory manifold. The corresponding Jacobian of the delay-output map then has two columns, associated with the phase coordinate and with the target variable. Extensions to multiple target variables are conceptually possible, but would require the corresponding increase in the compact-state dimension and of the number of independent sensitivity directions to be reconstructed.

Under the smoothness and observability assumptions stated in Section 2, the delay-embedding framework ensures that the compact state (ϑ,p) can be reconstructed from a finite stack of delayed output samples, provided that a suitable sampling period $T_{s}$ is selected. In the scalar-output case, Takens’s generic embedding bound gives m=2n+1 delayed samples. Since n=2 in the present setting, m=5 sampling instants are retained. It should be emphasized that the dimension entering this bound is the intrinsic dimension of the compact invariant set, and not the dimension of the full augmented state χ in (4). In the forced case the internal sensor state *x*, and hence the augmented state χ, may be high-dimensional; however, by the fiber-contraction property of Section 2 all trajectories asymptotically lose memory of their initial internal condition and collapse onto the attracting invariant graph, whose intrinsic dimension is $n=1+n_{p}$, equal to 2 in the present scalar-measurand setting. The embedding dimension is therefore governed by the dimension of this low-dimensional attracting set, on which the post-transient dynamics actually evolve, rather than by the dimension of the full transient or augmented state. The value m=5 should be interpreted as the generic scalar-output embedding bound associated with the compact state dimension n=2, rather than as an arbitrary tuning parameter. In the vector-output case, a smaller number of sampling instants may be sufficient for specific systems, because each instant provides *q* output components. Nevertheless, the same value m=5 is retained as a conservative baseline and as a common design choice across scalar and vector-valued outputs. The practical informativeness of the resulting delay vector also depends on the sampling time $T_{s}$, the excitation frequency, and the considered operating range. If $T_{s}$ is too small, consecutive samples are nearly identical, and the columns of the Jacobian of the delay map may become poorly independent. If $T_{s}$ is too large, the delay window increases and may become less compatible with the frozen-parameter approximation, while also increasing the risk of aliasing. A natural reference scale for this choice is the oscillation period of the phase variable ϑ, $T_{\vartheta }=2\pi /\omega$: the sampling time should be neither too small nor too large with respect to $T_{\vartheta }$, and can then be increased or decreased from this baseline according to the local conditioning analysis introduced below.

The resulting observation map is

(8) $$Y\left(t\right)=\left[\begin{array}{c} y(t)\\ y(t+T_{s})\\ y(t+2T_{s})\\ y(t+3T_{s})\\ y(t+4T_{s}) \end{array}\right]=H\left(\vartheta \left(t\right),p\right)=\left[\begin{array}{c} h(\vartheta (t),p)\\ h(\vartheta \left(t+T_{s}\right),p)\\ h(\vartheta \left(t+2T_{s}\right),p)\\ h(\vartheta \left(t+3T_{s}\right),p)\\ h(\vartheta \left(t+4T_{s}\right),p) \end{array}\right]\in R^{5q}$$

where each block $y\left(t+kT_{s}\right)=h\left(\vartheta \left(t+kT_{s}\right),p\right)$ belongs to $R^{q}$. Therefore, the scalar case q=1 leads to the usual five-dimensional delayed-output vector.

The map $H:X\subset R^{2}\to Y\subset R^{5q}$ is characterized numerically by repeated simulations of the sensor system, collecting pairs between the compact state and the corresponding delayed-output vector. Once this dataset is available, a neural-network function approximator is trained to approximate the inverse map $H^{-1}$, thus providing a direct, deadbeat-like reconstruction of the state from measured output samples. Although observability is assumed to be ensured by design, poor sensitivity of the delay vector to state variations may still degrade the numerical reconstruction. For this reason, a local invertibility analysis is introduced below.

### 3.1. Local Invertibility as a Necessary Condition

A necessary condition for local state reconstruction is the local invertibility of the observation map *H*. In differential terms, the Jacobian of *H* must have full column rank at every point x∈X. Its two columns represent the sensitivity of the delayed-output vector with respect to the phase coordinate and the target variable: if they are non-null and linearly independent, small variations along the two state directions produce distinguishable variations in the output manifold.

In practice, the Jacobian is approximated through finite differences: small perturbations δϑ and δp are applied to the phase coordinate and to the target variable, producing two numerical sensitivity vectors in the delay-output space. For an output dimension *q* and m=5 delayed sampling instants, the Jacobian has dimension 5q×2 and can be written as follows:

(9) $$J\left(\vartheta \left(t\right),p\right)=\frac{\partial H}{\partial x}\left(\vartheta \left(t\right),p\right)=\left[\begin{array}{cc} \frac{\partial H_{1}}{\partial \vartheta } & \frac{\partial H_{1}}{\partial p}\\ \frac{\partial H_{2}}{\partial \vartheta } & \frac{\partial H_{2}}{\partial p}\\ \frac{\partial H_{3}}{\partial \vartheta } & \frac{\partial H_{3}}{\partial p}\\ \frac{\partial H_{4}}{\partial \vartheta } & \frac{\partial H_{4}}{\partial p}\\ \frac{\partial H_{5}}{\partial \vartheta } & \frac{\partial H_{5}}{\partial p} \end{array}\right]\in R^{5q\times 2}$$

(10) $$H_{k}\left(\vartheta \left(t\right),p\right)=h\left(\vartheta \left(t+\left(k-1\right)T_{s}\right),p\right)\in R^{q}\;k=1,\ldots ,5$$

Each block row in (9) has dimension q×2. The adopted local observability indicator is the smallest eigenvalue of the Gramian matrix:

(11) $$\rho \left(\vartheta ,p\right)=\lambda_{\min}\left(J^{⊤}\left(\vartheta ,p\right)J\left(\vartheta ,p\right)\right)$$

A value ρ(ϑ,p)=0 indicates that the Jacobian is rank deficient and that the observation map is not locally invertible at the considered point. In practice, when *J* is computed numerically, small positive values signal poor local conditioning of the observation map, so that its local inversion becomes ill-posed at the considered point. It should be stressed that ρ is a necessary-condition indicator rather than a direct quantitative predictor of the achievable reconstruction error: a vanishing or near-vanishing ρ certifies that the chosen parameters and outputs surely produce a local loss of observability and must therefore be avoided or compensated by enriching the output map, whereas a value sufficiently far from zero only provides practical evidence of a locally well-conditioned map, without by itself guaranteeing a given reconstruction accuracy.

Evaluated numerically over the considered state domain, the indicator is not only diagnostic: it can also be used as a design criterion over admissible parameters, such as excitation frequency, component values, sampling time, and operating range, in order to select configurations that avoid nearly degenerate output manifolds.

The indicator ρ certifies only *local* invertibility and is therefore a necessary, but not sufficient, condition for reconstruction. A full-column-rank Jacobian guarantees that infinitesimally close states remain distinguishable, but it cannot detect a *global* ambiguity in which two states that are far apart in the compact domain are mapped to the same, or nearly the same, delayed-output stack.

Global injectivity could be probed directly through pairwise-distance arguments: over a representative sampling of the state domain, the distance

(12) $$∥H\left(x_{i}\right)-H\left(x_{j}\right)∥$$

in the delay-output space is compared with the separation of the corresponding states, and any pair of well-separated states yielding nearly coincident output vectors reveals an overlap between distinct branches of the delay-output manifold. Such overlaps are invisible to the local indicator and must be excluded or removed by enriching the output map, for the inverse to be well defined. A concrete instance arises in the MEMS benchmark of Section 4, where a sign symmetry of the raw capacitive readout maps opposite angular rates onto the same delayed-output stack; the output augmentation introduced there separates the two manifold branches and restores injectivity over the signed operating domain.

In practice, this pairwise test involves two nontrivial choices: the threshold below which two delayed-output stacks are declared indistinguishable, which should account for the expected measurement noise and for the local conditioning of the map, and the density of the state sampling, which must be fine enough to prevent overlaps between distinct branches from passing undetected between adjacent samples. In the present benchmark, the overlap is structural and, owing to the symmetry of the adopted state grid, the indistinguishable states fall on sampling nodes, so the test detects the overlap at essentially any resolution; a formal, resolution-aware criterion for generic configurations is left to future work.

### 3.2. Neural-Network Function Reconstruction

The final step consists of approximating the inverse map that recovers the compact state from the measured delay vector by training a neural-network function approximator on the collected state-output dataset. Radial Basis Function neural networks are well suited to this type of problem, because they approximate smooth nonlinear maps through localized units and can be trained effectively in low-dimensional settings [17,18]; in the present work, a feedforward architecture is used as a practical approximation tool for the inverse map. Feedforward networks are, moreover, universal function approximators [19]: any continuous map on a compact domain can be approximated to arbitrary accuracy, so that, whenever the achieved reconstruction accuracy is not satisfactory, it can in principle be improved by increasing the complexity of the network.

The network is trained on a sufficiently dense synthetic dataset, so as to capture the nonlinear structure of $H^{-1}$ while preserving generalization over the considered state domain; once calibrated, the learned map is used to estimate the state variables, in particular the target physical parameter *p*, from measured stacks of delayed output samples.

## 4. Application to a MEMS Vibratory Gyroscope

To assess the proposed indirect sensing framework on a micromechanical oscillating system, the methodology is applied to a Coriolis vibratory MEMS gyroscope [5,20,21], belonging to the class of externally forced oscillating sensors introduced in Section 2. Its response is generated by an actuated micro-oscillator with one drive mode and one orthogonal sense mode, while the target variable is the signed angular rate, denoted by Ω.

The operating principle is the following: the proof mass is forced to vibrate along the drive axis and, when the device is subjected to an angular rate about the sensitive axis, the Coriolis effect excites the orthogonal sense mode, so that the angular rate is not measured directly, but is encoded in the oscillatory response of the sense coordinate. To make the benchmark closer to a realistic MEMS transduction chain, the measured signal is not assumed to be the ideal sense displacement $x_{s}\left(t\right)$ but the nonlinear capacitive readout current generated by the variation in a parallel-plate sensing capacitance.

In this benchmark, the angular rate that is to be reconstructed is allowed to take both positive and negative values. As detailed below, this signed operating domain introduces an observability issue when only the raw current readout is considered: changing the sign of Ω reverses the Coriolis-induced sense response, but this effect can be compensated by a phase shift of π in the excitation angle ϑ, so that the reconstruction problem is affected not only by local sensitivity, but also by a possible global non-injectivity of the observation map.

### 4.1. Electromechanical Model and Raw Capacitive Readout

Let $x_{d}\left(t\right)$ denote the displacement along the drive axis and $x_{s}\left(t\right)$ the displacement along the sense axis. The MEMS gyroscope is modeled as a two-degree-of-freedom micro-oscillator, in which the drive motion is generated by electrostatic actuation and the sense motion is excited by the Coriolis coupling induced by the angular rate. Following standard lumped-parameter MEMS gyroscope models [20,21], the mechanical dynamics are written as

(13) $$m_{d}{\ddot{x}}_{d}\left(t\right)+c_{d}{\dot{x}}_{d}\left(t\right)+k_{d}x_{d}\left(t\right)=F_{d}\left(t\right)$$

(14) $$m_{s}{\ddot{x}}_{s}\left(t\right)+c_{s}{\dot{x}}_{s}\left(t\right)+k_{s}x_{s}\left(t\right)=-2m_{s}\Omega \;{\dot{x}}_{d}\left(t\right)+k_{q}x_{d}\left(t\right)$$

where $m_{d}$ and $m_{s}$ are the effective drive- and sense-mode masses, $c_{d}$ and $c_{s}$ are the viscous damping coefficients, $k_{d}$ and $k_{s}$ are the stiffness coefficients, $F_{d}\left(t\right)$ is the drive actuation force, and $k_{q}x_{d}\left(t\right)$ represents a possible parasitic quadrature or stiffness-coupling contribution. The Coriolis term $-2m_{s}\Omega {\dot{x}}_{d}\left(t\right)$ couples the drive velocity into the sense-mode dynamics.

In a capacitive MEMS gyroscope, the drive force can be generated by comb-finger electrostatic actuation. Assuming a biased drive voltage

(15) $$V_{d}\left(t\right)=V_{\mathrm{dc}}+v_{\mathrm{ac}}\left(t\right)$$

and retaining the useful first-order actuation term, the drive force is approximated as proportional to the AC component:

(16) $$F_{d}\left(t\right)=G_{d}\;v_{\mathrm{ac}}\left(t\right)$$

where $G_{d}$ is an electromechanical actuation gain depending on the comb-finger geometry, the dielectric permittivity, and the DC bias voltage.

The AC drive voltage is generated by the autonomous exosystem

(17) $$\dot{w}\left(t\right)=Sw\left(t\right)\;S=\left[\begin{array}{cc} 0 & -\omega_{0}\\ \omega_{0} & 0 \end{array}\right]\;v_{\mathrm{ac}}\left(t\right)=V_{\mathrm{ac}}Ew\left(t\right)\;E=\left[\begin{array}{cc} 1 & 0 \end{array}\right]$$

For ${∥w\left(0\right)∥}^{2}=1$, this gives

(18) $$w\left(t\right)=\left[\begin{array}{c} \cos\vartheta (t)\\ \sin\vartheta (t) \end{array}\right]\;\vartheta \left(t\right)=\omega_{0}t+\vartheta \left(0\right)\;v_{\mathrm{ac}}\left(t\right)=V_{\mathrm{ac}}\cos\vartheta \left(t\right)$$

The product between the electromechanical drive gain and the AC voltage amplitude is denoted by

(19) $$F_{0}=G_{d}V_{\mathrm{ac}}\;F_{d}\left(t\right)=F_{0}Ew\left(t\right)$$

Introducing the first-order state variables

(20) $$z\left(t\right)=\left[\begin{array}{c} z_{1}\left(t\right)\\ z_{2}\left(t\right)\\ z_{3}\left(t\right)\\ z_{4}\left(t\right) \end{array}\right]=\left[\begin{array}{c} x_{d}\left(t\right)\\ {\dot{x}}_{d}\left(t\right)\\ x_{s}\left(t\right)\\ {\dot{x}}_{s}\left(t\right) \end{array}\right]$$

the mechanical part of the gyroscope becomes

(21) $$\begin{array}{l} {\dot{z}}_{1}\left(t\right)=z_{2}\left(t\right) \end{array}$$

(22) $$\begin{array}{l} {\dot{z}}_{2}\left(t\right)=-\frac{c_{d}}{m_{d}}z_{2}\left(t\right)-\frac{k_{d}}{m_{d}}z_{1}\left(t\right)+\frac{F_{0}}{m_{d}}Ew\left(t\right) \end{array}$$

(23) $$\begin{array}{l} {\dot{z}}_{3}\left(t\right)=z_{4}\left(t\right) \end{array}$$

(24) $$\begin{array}{l} {\dot{z}}_{4}\left(t\right)=-\frac{c_{s}}{m_{s}}z_{4}\left(t\right)-\frac{k_{s}}{m_{s}}z_{3}\left(t\right)-2\Omega z_{2}\left(t\right)+\frac{k_{q}}{m_{s}}z_{1}\left(t\right) \end{array}$$

The angular rate Ω plays here the role of the target variable *p* of the general formulation and is treated through the frozen-parameter approximation of Section 2: it is not globally constant, but over each delay-embedding window it satisfies the slow-drift conditions, so that the reduced model is written locally as $\dot{\Omega }\left(t\right)=0$.

The raw electromechanical readout is constructed from the capacitive sense transducer. The sense displacement changes the gap of a parallel-plate capacitance, modeled as

(25) $$C_{s}\left(x_{s}\right)=\frac{\varepsilon_{0}\varepsilon_{r}n_{s}w_{p}h}{g_{s}+x_{s}\left(t\right)}$$

where $\varepsilon_{0}$ is the vacuum permittivity, $\varepsilon_{r}$ is the relative permittivity, $n_{s}$ is the number of sensing plates, $w_{p}$ is the plate overlap length, *h* is the device thickness, and $g_{s}$ is the nominal sense gap. The admissible motion is assumed to satisfy $g_{s}+x_{s}\left(t\right)>0$ so that the capacitance remains well defined.

Under a constant sensing bias voltage $V_{\mathrm{off}}$, the capacitive readout current is as follows:

(26) $$i_{s}\left(t\right)=V_{\mathrm{off}}\frac{dC_{s}}{dt}=-V_{\mathrm{off}}\varepsilon_{0}\varepsilon_{r}n_{s}w_{p}h\frac{{\dot{x}}_{s}\left(t\right)}{{\left(g_{s}+x_{s}\left(t\right)\right)}^{2}}$$

(27) $$i_{s}\left(t\right)=-K_{\mathrm{cap}}\frac{z_{4}\left(t\right)}{{\left(g_{s}+z_{3}\left(t\right)\right)}^{2}}\;K_{\mathrm{cap}}=V_{\mathrm{off}}\varepsilon_{0}\varepsilon_{r}n_{s}w_{p}h$$

Since a constant readout gain only rescales the measured signal and does not affect the injectivity properties of the delay map, the capacitive readout is normalized by the nominal gap. The raw normalized current output is therefore defined as

(28) $$y_{c}\left(t\right)=-g_{s}^{2}\frac{z_{4}\left(t\right)}{{\left(g_{s}+z_{3}\left(t\right)\right)}^{2}}$$

By stacking the mechanical state and the excitation state, the dynamic sensor-excitation state is written as

(29) $$\xi \left(t\right)=\left[\begin{array}{c} z(t)\\ w(t) \end{array}\right]$$

whereas the angular rate Ω is treated as an additional frozen parameter over the reconstruction window, consistently with the frozen-parameter approximation introduced above. Equivalently, one may define the parameter-augmented state

(30) $$\xi_{\Omega }\left(t\right)=\left[\begin{array}{c} z(t)\\ w(t)\\ \Omega (t) \end{array}\right]\;\dot{\Omega }\left(t\right)=0$$

At steady state, the mechanical variables converge to an attractive oscillatory response parametrized by the forcing phase and by the angular rate. Consequently, in analogy with the general framework introduced in Section 2 and Section 3, the effective compact state used for delay-embedding reconstruction is x=(ϑ,Ω), where ϑ denotes the forcing phase and Ω is the signed angular rate to be reconstructed.

### 4.2. Raw-Output Non-Observability and Augmented Output Map

For analysis, the scalar raw-output delay map is first defined as

(31) $$y_{c}\left(t\right)=\left[\begin{array}{c} y_{c}\left(t\right)\\ y_{c}\left(t+T_{s}\right)\\ y_{c}\left(t+2T_{s}\right)\\ y_{c}\left(t+3T_{s}\right)\\ y_{c}\left(t+4T_{s}\right) \end{array}\right]=H_{c}\left(\vartheta \left(t\right),\Omega \right)$$

with $H_{c}:X\subset R^{2}\to R^{5}$. The corresponding Jacobian is the following:

(32) $$J_{c}\left(\vartheta ,\Omega \right)=\frac{\partial H_{c}}{\partial x}\left(\vartheta ,\Omega \right)=\left[\begin{array}{cc} \frac{\partial H_{c}}{\partial \vartheta } & \frac{\partial H_{c}}{\partial \Omega } \end{array}\right]\in R^{5\times 2}$$

Its numerical approximation is exploited to assess local invertibility through the indicator $\rho_{c}\left(\vartheta ,\Omega \right)$ associated with the mapping $H_{c}$.

If the normalized current signal (28) is used as the only measured output, two distinct observability issues arise over a signed angular-rate range. The first one is a local loss of information around Ω=0. Indeed, by neglecting the parasitic input term in (14), the sense response is driven only by the Coriolis contribution, whose amplitude is proportional to Ω. Therefore, when Ω=0, the raw capacitive output loses the phase-dependent sense response, and the forcing phase ϑ cannot be reconstructed from $y_{c}$ alone. This degeneracy is also visible through the local Jacobian, since the delayed-output map becomes poorly sensitive to variations of the compact state near the sign change in Ω.

The second issue is instead a global non-injectivity of the raw-output delay map. For nonzero angular rates, changing the sign of Ω reverses the sign of the Coriolis-induced sense oscillation. However, the same output sequence can be generated by shifting the forcing phase by π, leading to the symmetry $y_{c}\left(\vartheta ,\Omega \right)=y_{c}\left(\vartheta +\pi ,-\Omega \right)$. As a consequence, two states that are far apart in the compact state domain may generate identical delayed-output stacks. This ambiguity is not necessarily detected by a purely local analysis, because the indistinguishable states are generally not infinitesimally close.

In vibratory gyroscopes, this type of ambiguity is commonly handled through coherent demodulation, which requires a phase-recovery or phase-reference circuit synchronized with the drive motion and provides the phase information needed to distinguish the sign of the angular rate and to recover Ω from the measured oscillatory response. In the present delay-embedding framework, the same requirement can be interpreted as the need to enrich the output map so that the inverse reconstruction is well defined over the whole (ϑ,Ω) domain. Rather than replacing the raw capacitive readout with a pre-demodulated signal, the measured output is augmented: the first component is the raw normalized capacitive current output $y_{c}\left(t\right)$, while the second component is the known AC drive voltage $v_{\mathrm{ac}}\left(t\right)$, which is phase-synchronized with the capacitive readout:

(33) $$y_{a}\left(t\right)=\left[\begin{array}{c} y_{c}\left(t\right)\\ v_{\mathrm{ac}}\left(t\right) \end{array}\right]=\left[\begin{array}{c} -g_{s}^{2}\frac{z_{4}\left(t\right)}{{\left(g_{s}+z_{3}\left(t\right)\right)}^{2}}\\ V_{\mathrm{ac}}\cos\vartheta \left(t\right) \end{array}\right]$$

Since $v_{\mathrm{ac}}\left(t\right)=V_{\mathrm{ac}}\cos\vartheta \left(t\right)$ is already available from the drive electronics, the augmented output explicitly provides the phase information needed to remove the sign ambiguity without introducing an additional measured mechanical quantity.

The augmented delay vector is then constructed by concatenating the five bidimensional output samples:

(34) $$y_{a}\left(t\right)=\left[\begin{array}{c} y_{c}\left(t\right)\\ v_{\mathrm{ac}}\left(t\right)\\ y_{c}\left(t+T_{s}\right)\\ v_{\mathrm{ac}}\left(t+T_{s}\right)\\ y_{c}\left(t+2T_{s}\right)\\ v_{\mathrm{ac}}\left(t+2T_{s}\right)\\ y_{c}\left(t+3T_{s}\right)\\ v_{\mathrm{ac}}\left(t+3T_{s}\right)\\ y_{c}\left(t+4T_{s}\right)\\ v_{\mathrm{ac}}\left(t+4T_{s}\right) \end{array}\right]=H_{a}\left(\vartheta \left(t\right),\Omega \right)$$

Thus, the neural inverse map receives a ten-dimensional input vector, while the compact state to be reconstructed remains two-dimensional:

(35) $$H_{a}:X\subset R^{2}\to R^{10}\;H_{a}^{-1}:R^{10}\to R^{2}$$

The corresponding Jacobian and local observability indicator are

(36) $$J_{a}\left(\vartheta ,\Omega \right)=\frac{\partial H_{a}}{\partial x}\left(\vartheta ,\Omega \right)=\left[\begin{array}{cc} \frac{\partial H_{a}}{\partial \vartheta } & \frac{\partial H_{a}}{\partial \Omega } \end{array}\right]\in R^{10\times 2}$$

(37) $$\rho_{a}\left(\vartheta ,\Omega \right)=\lambda_{\min}\left(J_{a}^{⊤}\left(\vartheta ,\Omega \right)J_{a}\left(\vartheta ,\Omega \right)\right)$$

Compared with the raw scalar output, the augmented output includes direct information on the drive phase and is therefore designed to address the global sign ambiguity affecting the raw current readout.

The disambiguation provided by the augmented map relies on $v_{\mathrm{ac}}\left(t\right)=V_{\mathrm{ac}}\cos\vartheta \left(t\right)$ being phase-synchronized with the capacitive readout. Since the drive-reference and sense channels are acquired through distinct electronic paths, a residual phase offset Δϑ between them may be present in practice. A constant offset shifts the apparent drive phase and, because the recovery of the angular-rate sign rests on the relative phase between the two channels, a sufficiently large or slowly drifting Δϑ can partially re-introduce the very sign ambiguity that the augmentation is meant to remove. The reconstruction is thus expected to tolerate small and stable phase errors, which can be absorbed by calibration or learned by training the inverse map over a range of reference offsets, while still requiring adequate synchronization of the reference channel; a quantitative assessment of this tolerance is left to future work.

### 4.3. Data Generation and Neural Reconstruction in Ideal Conditions

For the MEMS gyroscope benchmark, the synthetic dataset is generated over the rectangular state domain defined by the forcing phase and the signed angular rate:

(38) $$\vartheta \in \left[0,2\pi \right)\;\Omega \in \left[\Omega_{\min},\Omega_{\max}\right]=\left[-6,\;6\right]\;\mathrm{rad}/s$$

A uniform N×N grid is selected over this domain, with $N_{\vartheta }=N_{\Omega }=N$, yielding $N_{\mathrm{states}}=N^{2}$ state-output pairs for training. Since the density of the training grid is a key hyperparameter of the proposed approach, three grid sizes are considered, N∈{100,150,200}, and the resulting networks are first compared in ideal conditions in order to select a reference configuration for the subsequent analysis under output noise. For each state (ϑ,Ω), the corresponding delayed output stack is generated in the steady-state oscillatory regime, and the nonlinear readout (28) is evaluated algebraically at the corresponding delayed phases.

The finite-difference perturbations used to compute the Jacobian-based local observability indicators are selected according to a grid-relative criterion.

(39) $$\delta \vartheta =\eta \frac{2\pi }{N_{\vartheta }}\;\delta \Omega =\eta \frac{\Omega_{\max}-\Omega_{\min}}{N_{\Omega }}\;\eta =10^{-3}$$

The raw-output map $H_{c}$ and the augmented-output map $H_{a}$ are evaluated over the same state grid, so that the effect of adding $v_{\mathrm{ac}}$ can be assessed directly.

The fixed physical and operating parameters adopted for the MEMS benchmark are summarized in Table 1. The values define a representative numerical configuration inspired by lumped-parameter and envelope models of capacitive MEMS gyroscopes [20,21], rather than the exact reproduction of a specific fabricated device. The sense gap is taken consistently with the geometric data reported in [20], while the modal frequencies and quality factors are selected within a realistic weakly damped split-mode operating regime and are used to compute the equivalent stiffness and damping coefficients. The quadrature coefficient is set to zero in order to isolate the Coriolis-induced contribution.

The sampling time was selected with respect to the excitation period, rather than as an independent arbitrary parameter. In the adopted MEMS configuration, the excitation frequency is $\omega_{0}=2\pi f_{d}$, with $f_{d}=3535\;\mathrm{Hz}$. Therefore, the excitation period is $T_{0}≃282.9\;\mu s$. The selected value $T_{s}=20\;\mu s$ gives

(40) $$\frac{T_{s}}{T_{0}}≃0.0707$$

corresponding to approximately 14.1 sampling intervals per excitation period. Equivalently, the phase advance between two consecutive delayed samples is $\omega_{0}T_{s}≃0.444\;\mathrm{rad}≃25.45{}^{\circ}$. Thus, consecutive delayed samples are sufficiently separated in phase to provide non-redundant information on the oscillatory response, while still being collected over a short time interval. Since m=5, the total delay-embedding window is

(41) $$T_{\mathrm{emb}}=\left(m-1\right)T_{s}=80\;\mu s≃0.283T_{0}$$

which spans approximately 101.8° of the excitation cycle. This provides a compromise between informativeness of the delayed-output stack and compatibility with the frozen-angular-rate approximation over each reconstruction window. This criterion also indicates that, if the excitation frequency or operating range were changed, the sampling time should be re-evaluated.

**Figure 1:**
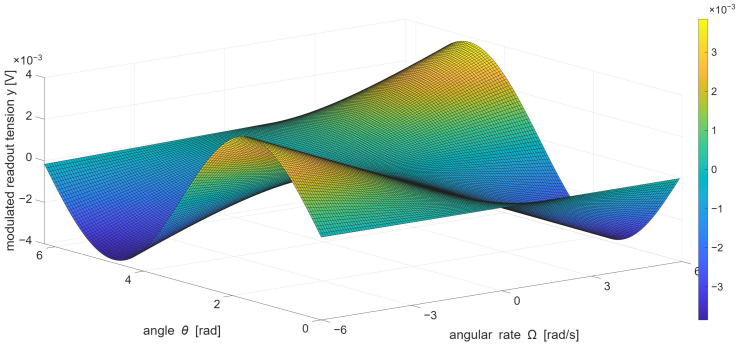
First sampled value of the raw normalized capacitive readout over the MEMS state rectangle. The surface corresponds to $y_{c}\left(\vartheta ,\Omega \right)$ before output augmentation.

As a first qualitative check, the first sampled value of $y_{c}\left(t\right)$ is plotted over the state rectangle (ϑ,Ω) (Figure 1). The surface vanishes around Ω=0 and changes sign consistently with the signed Coriolis excitation; by itself, however, it does not remove the global ambiguity between opposite angular rates and phase-shifted trajectories.

**Figure 2:**
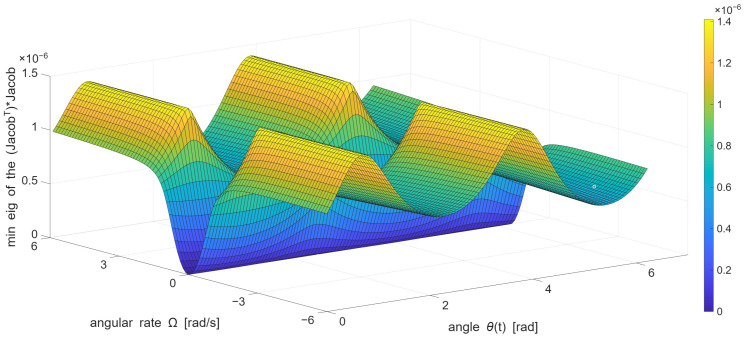
Local observability indicator $\rho_{c}\left(\vartheta ,\Omega \right)=\lambda_{\min}\left(J_{c}^{⊤}J_{c}\right)$ for the raw scalar MEMS output stack. The raw output stack has a dimension of 5.

The local observability indicator $\rho_{c}\left(\vartheta ,\Omega \right)$ for the raw scalar stack is then evaluated (Figure 2). The resulting surface highlights the loss of rank where the angular rate changes sign, confirming that the raw-output map is locally ill-conditioned near Ω=0; away from this region, the map is locally well conditioned, yet the global sign ambiguity still persists.

**Figure 3:**
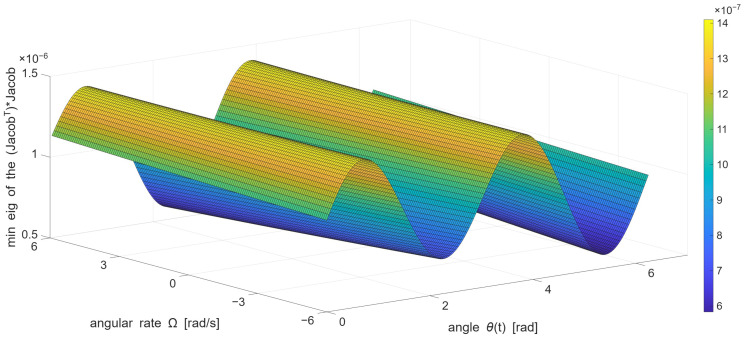
Local observability indicator $\rho_{a}\left(\vartheta ,\Omega \right)=\lambda_{\min}\left(J_{a}^{⊤}J_{a}\right)$ for the augmented MEMS output stack. The augmented output stack has dimension 10 and alternates $y_{c}$ and $v_{\mathrm{ac}}$.

The same analysis is repeated for the augmented output stack (34) (Figure 3). In this case, the Jacobian has dimensions of 10×2 since each of the five delayed samples contains both the current output and the known AC drive voltage. The resulting indicator shows the improved local conditioning obtained by adding the drive-reference channel to the measured output. However, this local full-rank property remains a necessary, but not sufficient, condition for global invertibility of the observation map over the whole (ϑ,Ω) domain.

Because the observation map is available pointwise on the sampling grid, the global-injectivity test of Section 3 can be carried out directly: for every pair of well-separated grid states the distance $∥H\left(x_{i}\right)-H\left(x_{j}\right)∥$ between the corresponding delayed-output stacks is evaluated, and any nearly coincident pair is flagged as a manifold overlap, while states closer than a few grid steps in either ϑ or Ω are excluded so that only genuinely distinct states are compared. Carried out on the (ϑ,Ω) grid, this test sharply separates the two output choices. For the augmented ten-dimensional output, no well-separated pair produces a coincident stack: the number of such matches is zero over the entire grid, and the minimum pairwise distance between well-separated states remains strictly positive, of the order of $10^{-4}$. For the raw five-dimensional output, instead, the same test returns for essentially every state exactly one well-separated partner with a coincident output stack, rising to two partners in the neighborhood of ϑ=π, while the minimum pairwise distance collapses to the numerical floor, of the order of $10^{-18}$. This quantitatively confirms the manifold overlap induced by the sign symmetry $y_{c}\left(\vartheta ,\Omega \right)=y_{c}\left(\vartheta +\pi ,-\Omega \right)$ and shows that the output augmentation removes it by separating the two manifold branches over the signed operating domain.

After the observability assessment, the augmented state-output pairs are used to train a neural approximator of the inverse map. The network input is the ten-dimensional delayed vector $y_{a}\left(t\right)$, while the target output is the compact state (ϑ,Ω).

For reproducibility, the neural-network implementation details are specified as follows. The inverse map is approximated in MATLAB by a fully connected feedforward neural network implemented through feedforwardnet ([50,20,10]), i.e., three hidden layers with 50, 20, and 10 neurons; the hidden-layer transfer functions are set to radbas and the output layer uses purelin. Training relies on the Levenberg–Marquardt backpropagation algorithm (trainlm) with the mean squared error (mse) as the performance function, while input and output preprocessing use the standard MATLAB functions associated with feedforwardnet, including mapminmax scaling. The adopted configuration is motivated as follows. For the smooth function-approximation and regression task considered here, the Levenberg–Marquardt backpropagation algorithm is among the fastest and most reliable training methods for small- and medium-sized networks, which is the regime of the present architecture; the corresponding MATLAB default (trainlm) was therefore retained rather than replaced by a generic first-order optimizer. The architecture itself was not the result of an exhaustive search: a target reconstruction accuracy was prescribed in terms of an RMSE threshold, and the reported configuration was retained because it met this specification on the validation set. The random seed was set to 1 before network initialization, so that the reported metrics are reproducible across training runs.

For each training grid, the corresponding network is assessed on a separate validation set that does not share any state with the training data. In contrast with a validation grid that reuses the training phase values, here the validation states are taken at the midpoints between every pair of consecutive training samples in both coordinates, so that no validation point lies on a training node along either the phase or the angular-rate axis. For a training grid of size N×N this yields a validation grid of (N−1)×(N−1) points, each located at the center of a training cell, i.e., at the point farthest from the surrounding training nodes and hence the most demanding location for interpolation within the operating range. Denoting by $\vartheta_{i}^{\mathrm{tr}}$ and $\Omega_{i}^{\mathrm{tr}}$ the training grids, the validation samples are

(42) $$\vartheta_{i}^{\mathrm{val}}=\frac{\vartheta_{i}^{\mathrm{tr}}+\vartheta_{i+1}^{\mathrm{tr}}}{2},\;\Omega_{j}^{\mathrm{val}}=\frac{\Omega_{j}^{\mathrm{tr}}+\Omega_{j+1}^{\mathrm{tr}}}{2},\;i,j=1,\ldots ,N-1$$

and the full validation set is the Cartesian product of these midpoint values. This is a stricter generalization test than evaluating the network off-grid along the angular rate only, since every validation state is now genuinely unseen in both coordinates.

The separation between validation and training states is quantified through the nearest-neighbor distance in normalized compact-state coordinates. Because the validation samples are cell-center midpoints, the nearest distance from the training grid is one half of the training step along each coordinate, so that, unlike a phase-on-grid validation, the phase distance is now strictly positive. For the adopted 150×150 grid this gives $d_{\vartheta ,\mathrm{NN}}≃2.09\cdot 10^{-2}\;\mathrm{rad}$, $d_{\Omega ,\mathrm{NN}}=4.0\cdot 10^{-2}\;\mathrm{rad}/s$ and $d_{\mathrm{norm},\mathrm{NN}}≃4.71\cdot 10^{-3}$.

**Figure 4:**
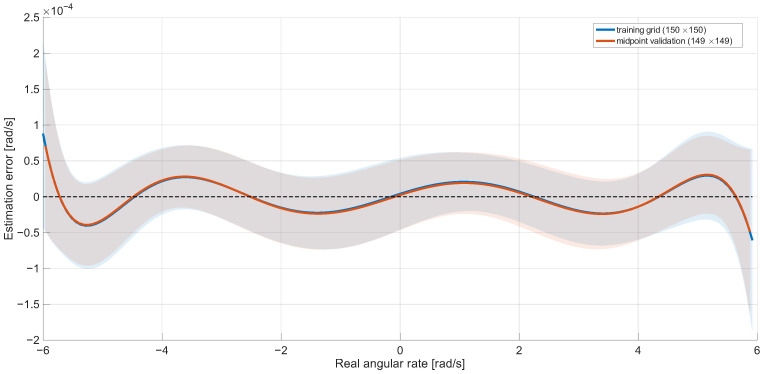
Angular -rate estimation error as a function of the true signed angular rate, for the adopted 150×150 network. Two curves are shown: the reconstruction error evaluated on the training grid (150×150) and on the off-grid midpoint validation set (149×149). For each curve, the solid line is the mean of the error over the phase coordinate at each angular-rate value, and the shaded band denotes ±1 standard deviation over the phase. The network is trained on the augmented output stack containing the raw capacitive readout and the AC drive reference.

Table 2 reports the ideal-conditions accuracy for the three training densities. On the coarsest grid (N=100) the validation error exceeds the training error by more than two orders of magnitude (ratio ≈190): the network fits the training nodes accurately but interpolates poorly between them, so the 100×100 sampling is too coarse to constrain the inverse map over the cell interiors. Increasing the density closes this gap rapidly. At N=150, the validation RMSE drops to $8.0\cdot 10^{-5}\;\mathrm{rad}/s$ with a validation-to-training ratio of 1.74 and at N=200 to $4.3\cdot 10^{-5}\;\mathrm{rad}/s$ with a ratio of 1.09, i.e., an essentially closed generalization gap. The error-versus-density behavior thus exhibits a marked knee at N=150, where most of the accuracy improvement is already attained, whereas moving to N=200 only halves the RMSE at the cost of a substantially larger training set, which grows as $N^{2}$. For this reason, the 150×150 network is selected as the reference configuration for the subsequent analysis under output noise: it delivers a near-saturated generalization accuracy, of the order of $10^{-4}\;\mathrm{rad}/s$, at a moderate dataset size. This confirms that the augmented delay-embedding representation provides enough information to reconstruct the signed angular rate directly from the measured output stack (Figure 4).

These results show that the proposed delay-embedding and neural inverse-learning workflow can be effectively applied to a micromechanical angular-rate sensor, the key additional step being the construction of an output map that preserves the information needed to distinguish signed angular rates, here achieved by augmenting the nonlinear current output with the known AC drive voltage.

### 4.4. Robustness Assessment Under Output Noise

The previous validation was performed under ideal conditions, using delayed-output stacks generated from the same nominal MEMS model adopted for training. To further assess the practical robustness of the proposed reconstruction procedure, an additional test is introduced, in which the neural network is kept fixed after being trained on the nominal noise-free dataset: the observed degradation is therefore not compensated by retraining and directly reflects the sensitivity of the learned inverse map to non-ideal validation data. Retraining on noisy data could, in principle, further robustify the reconstruction, but is left to future work, since it would require substantially larger and more representative training datasets.

The first test evaluates the effect of additive measurement noise on the capacitive readout. As in the ideal case, each reconstruction relies on a single delayed-output stack of five samples, which spans only 80μs and therefore provides one instantaneous estimate of the angular rate, intrinsically sensitive to the noise affecting the current readout. However, since the angular rate is, by assumption, slowly varying with respect to the drive oscillation, consecutive instantaneous estimates can be combined over time to attenuate the noise without significantly affecting the reconstructed signal. This is obtained by post-filtering the sequence of reconstructed angular rates with a first-order low-pass filter, rather than by modifying the trained network. Both this post-filtering stage and the low-pass stage of coherent demodulation rest on the same underlying principle: they attenuate the high-frequency measurement noise by exploiting the assumption that the quantity of interest, here the slowly varying angular rate Ω, is band-limited, and both therefore limit the achievable bandwidth. If anything, the comparison is slightly less favorable to the present approach: in coherent demodulation the noise acts on the measured signal and its effect on the estimate is transparent, whereas here the way the measurement noise on the current readout is remapped onto the reconstructed state by the nonlinear network is not known a priori; the cut-off frequency cannot therefore be derived in closed form and must be selected empirically, as a compromise between noise rejection and preserved bandwidth.

To reproduce a realistic time evolution, the validation is organized into independent runs. In each run the angular rate Ω is held constant while the drive phase ϑ evolves in time. A new angular-rate estimate is produced from each non-overlapping window of five samples, i.e., every $T_{\mathrm{est}}=5T_{s}=100\;\mu s$, and $N_{\mathrm{run}}=150$ consecutive estimates are collected per run. One run is generated for each of the 149 validation angular rates of the adopted 150×150 configuration. As in the ideal validation, the white Gaussian perturbation is applied only to the current-readout entries, while the drive-reference entries are kept noise-free, since $v_{\mathrm{ac}}\left(t\right)$ is generated by the excitation electronics and acts as a synchronization signal:

(43) $${\tilde{y}}_{c}\left(t+kT_{s}\right)=y_{c}\left(t+kT_{s}\right)+\varepsilon_{k},\;\varepsilon_{k}∼N\left(0,\sigma_{y}^{2}\right),\;k=0,\ldots ,4$$

The noise standard deviation $\sigma_{y}$ is set to a conservative 0.5% RMS perturbation of the current output, as typical values are around 0.2%.

Let $\hat{\Omega }\left[k\right]$ denote the sequence of instantaneous angular-rate estimates produced along a run. The filtered estimate $\bar{\Omega }\left[k\right]$ is obtained through the first-order low-pass filter

(44) $$\bar{\Omega }\left[k\right]=\bar{\Omega }\left[k-1\right]+\alpha \left(\hat{\Omega }\left[k-1\right]-\bar{\Omega }\left[k-1\right]\right),\;\alpha =\frac{T_{\mathrm{est}}}{\tau }=2\pi f_{c}\;T_{\mathrm{est}}$$

obtained by forward-Euler discretization of the continuous-time first-order differential equation $\tau \dot{\bar{\Omega }}+\bar{\Omega }=\hat{\Omega }$, with time constant $\tau =1/(2\pi f_{c})$ and cut-off frequency $f_{c}$. The cut-off is a design parameter: it must be high enough to preserve the bandwidth of the angular-rate signal of interest and low enough to suppress the readout noise. The filter is initialized at zero, so that the first samples of each run describe the start-up transient. The reconstruction accuracy is then evaluated on the steady-state portion of each run, namely the last $N_{\mathrm{tail}}=50$ filtered samples.

**Figure 5:**
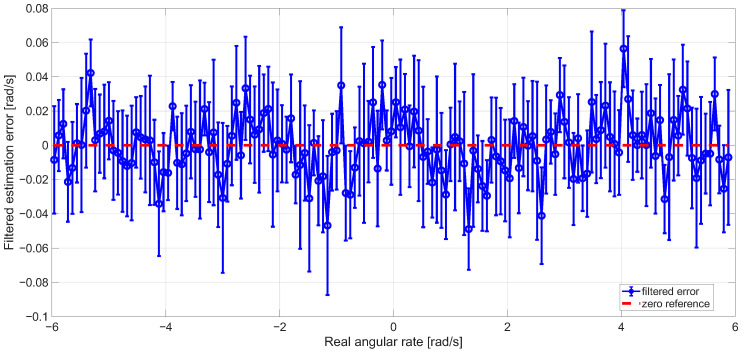
Steady -state filtered reconstruction error as a function of the true signed angular rate ($f_{c}=200\;\mathrm{Hz}$), for the adopted 150×150 network. Markers and error bars denote the mean and the standard deviation of the error over the last $N_{\mathrm{tail}}=50$ filtered samples of each run.

For each run—that is, for each of the 149 validation angular rates—the root-mean-square error (RMSE) is evaluated on the steady-state portion defined above, together with its normalized counterpart (NRMSE), obtained by dividing the per-run RMSE by the corresponding true angular rate. Runs with |Ω|≤0.5rad/s are excluded from the NRMSE statistics, since the normalization is ill-defined near Ω=0. A Monte Carlo validation with $N_{\mathrm{MC}}=30$ independent noise realizations is performed; the mean and the standard deviation of each metric over the realizations are reported separately. The same noise realizations are reused across all cut-off frequencies, so that the effect of $f_{c}$ can be compared fairly. The results are summarized in Table 3; the corresponding steady-state error profile across the validation angular rates at $f_{c}=200\;\mathrm{Hz}$ is shown in Figure 5.

Without post-filtering, the instantaneous per-window estimate yields an RMSE of about $1.33\cdot 10^{-1}\;\mathrm{rad}/s$ (≈7.6°/s). The low-pass post-filtering reduces this error substantially, and the residual error decreases monotonically as the cut-off is lowered, trading angular-rate bandwidth for noise rejection (Table 3). At the representative operating point $f_{c}=200\;\mathrm{Hz}$, which preserves a wide angular-rate bandwidth, the filtered RMSE is $3.39\cdot 10^{-2}\;\mathrm{rad}/s$ (≈1.94°/s), with a normalized error of the order of 1.5%. A lower cut-off can be adopted whenever a narrower angular-rate bandwidth is acceptable, at the cost of a slower response. The adopted cut-off values are also consistent with the angular-rate bandwidth typically required in MEMS-gyroscope applications: although vibratory gyroscopes can provide output data rates on the order of 1kHz, the useful measurement bandwidth in common motion-sensing devices is usually confined to the 100–200Hz range [22].

The residual filtered error is characterized by two contributions of very different magnitude. First, the intrinsic imperfection of the learned inverse map: applying the same post-filtering pipeline to the noise-free runs yields a filtered RMSE of only about $2.6\cdot 10^{-5}\;\mathrm{rad}/s$ (maximum deviation $7.9\cdot 10^{-5}\;\mathrm{rad}/s$), which reflects the accuracy of the reconstruction at phase values not contained in the training grid. Since this noise-free floor lies more than three orders of magnitude below the RMSE obtained under noise, the filtered error is entirely dominated by the measurement noise rather than by the reconstruction itself. Second, the noise contribution does not fully vanish under low-pass filtering: because the inverse map is nonlinear, a zero-mean perturbation of the output does not produce a zero-mean angular-rate error, so a velocity-dependent bias remains, of the order of $1.4\cdot 10^{-2}\;\mathrm{rad}/s$ on average, that a linear filter cannot remove. These observations indicate that, while simple post-filtering already brings the reconstruction error to the same order of magnitude as specialized model-based estimators, a further reduction would require a model-based recursive estimator, such as a Kalman filter exploiting the delayed-output stack as a noisy full-state measurement, or a re-training of the inverse map directly on noisy data.

It is instructive to relate these figures to dedicated model-based estimators for vibratory gyroscopes. In [23], for instance, the rotation rate of a parametrically excited micro gyroscope is reconstructed through a two-stage Extended/Unscented Kalman filtering scheme, attaining an angular-rate error of the same order of magnitude as the one obtained here after post-filtering. A direct quantitative comparison is difficult, since the two studies rely on different sensor configurations, excitation schemes and noise conditions: the reference is therefore qualitative, and a specialized estimator, explicitly tailored to a specific device and its excitation, could potentially reach a somewhat lower error for that configuration.

### 4.5. Real-Time Feasibility for Embedded Deployment

Although neural-network-based methods are often perceived as computationally demanding, this limitation is unlikely to hinder the proposed approach in the case of MEMS gyroscope sensors. According to recent surveys, feedforward neural networks (FNNs) implemented on FPGA platforms achieve inference times of the order of 1μs per sample, even for relatively complex high-precision architectures [24]. Since the proposed model also belongs to the FNN class, comparable inference latencies are expected, although the exact execution time depends on the network architecture and on the hardware implementation.

For MEMS gyroscope applications, typical sampling frequencies lie in the kHz range. Consequently, the time available for processing each sample exceeds the reported inference latency by several orders of magnitude, indicating that online deployment is feasible without sample decimation or any reduction in the sensor data rate. While an implementation-specific benchmark is beyond the scope of this work, the computational complexity of the proposed method does not appear to constitute a practical obstacle to real-time operation in the considered application domain.

## 5. Conclusions

This paper proposed a delay-embedding procedure for indirect sensing in oscillating and periodically forced sensors. The sensor and its excitation are modeled as a single autonomous system, in which the measurand is a slowly varying state over the reconstruction window, and the excitation is described by a phase variable; delayed output samples then define an observation map whose inverse is approximated by a feedforward neural network. With respect to the scalar-output conference prequel, the formulation was extended to vector-valued outputs, allowing additional channels or known reference signals to be exploited. The method was applied to a vibratory MEMS gyroscope for signed angular-rate reconstruction, a micromechanical sensor with nonlinear electromechanical transduction in which the raw capacitive current alone does not yield a well-posed problem over a signed domain, since opposite angular rates become indistinguishable after a phase shift in the excitation. This was resolved by augmenting the output with the known AC drive reference, which supplies the missing phase information without an additional mechanical measurement.

The Jacobian-based indicator $\lambda_{\min}\left(J^{⊤}J\right)$ served as a local necessary condition: the augmented map proved better conditioned than the raw one, and in ideal conditions the learned inverse reconstructed the signed angular rate with small errors. A preliminary robustness assessment was also carried out: under additive white Gaussian noise on the current channel, the instantaneous per-window estimate is perturbed, but since the angular rate is slowly varying, a first-order low-pass post-filtering of the reconstructed sequence brings the error back to the order of magnitude of specialized model-based estimators.

The objective of the proposed method is complementary to that of dedicated model-based estimators: rather than maximizing accuracy for a single device, it provides a common reconstruction workflow that transfers across heterogeneous oscillating sensors, does not require tuning of process- and measurement-noise covariance matrices, and can in principle reconstruct additional internal states or physical parameters from the same embedded representation.

Throughout this study, the sensor model has been assumed to be accurately known, and robustness to parametric uncertainty has been deliberately left outside the scope of the analysis. This choice mirrors the established practice for vibratory MEMS gyroscopes, where parameter deviations induced by fabrication tolerances, aging, and environmental conditions are a well-known and nontrivial issue: they are conventionally handled either through dedicated calibration procedures, which identify or compensate the actual device behavior, or through adaptive and observer-based schemes that estimate the uncertain parameters online. The latter, however, are subject to an observability requirement which, in the adaptive literature, takes the form of a persistent-excitation condition, enforced by specific control and parameter update laws [25]. The proposed framework offers a natural route to the same goal: since the delay-embedding formulation can host additional slowly varying states, drifting physical parameters can in principle be appended to the compact state and estimated jointly with the measurand. Exploiting this potentiality is left to future work and requires a preliminary observability verification: depending on the admissible parameter range, local observability may either hold natively under the present single-frequency interrogation or need to be enforced by enriching the exosystem signal that excites the sensor with additional frequency components to restore the persistent-excitation requirement of adaptive schemes. As for the measurand itself, this local verification is necessary but not sufficient, since the practical reconstructability of the augmented inverse also rests on the global injectivity of the delay-output map.

Future work could proceed along four directions. First, global injectivity can be investigated more systematically through pairwise-distance or manifold-overlap analyses, complementing the local indicator; in the present work, threshold identification and grid-point density still need to be formally addressed. Second, the influence of sampling time, embedding dimension, excitation frequency, and reference-channel synchronization needs further study. Third, to address noise robustness, more advanced estimation schemes could be used, such as a Kalman filter exploiting the delayed-output stack as a noisy full-state measurement, or a re-training of the inverse map on noisy data, to further reduce the residual error and recover the full internal state. Fourth, the method should be validated on higher-fidelity simulations and, ultimately, on experimental sensing chains.

## Figures and Tables

**Table 1:** Representative physical and operating parameters used for the MEMS gyroscope benchmark.

Parameter	Unit	Value
$m_{d}=m_{s}$	kg	$0.89\cdot 10^{-6}$
$f_{d}$	Hz	3535
$f_{s}$	Hz	3554
$Q_{d}$	-	22,320
$Q_{s}$	-	24,195
$k_{d}$	N/m	439.06
$k_{s}$	N/m	443.80
$c_{d}$	N s/m	$8.86\cdot 10^{-7}$
$c_{s}$	N s/m	$8.21\cdot 10^{-7}$
$k_{q}$	N/m	0
$F_{0}$	N	$6.79\cdot 10^{-8}$
$g_{s}$	m	$9.46\cdot 10^{-6}$
$V_{\mathrm{ac}}$	V	1
$\omega_{0}$	rad/s	$2\pi f_{d}$
$T_{s}$	s	$20\cdot 10^{-6}$
*m*	-	5

**Table 2:** Ideal-conditions angular-rate reconstruction accuracy versus training-set density. Each network is trained on an N×N grid and evaluated on the (N−1)×(N−1) off-grid midpoint validation set. ${\mathrm{RMSE}}_{\mathrm{tr}}$ is the error on the training grid and the last column is the validation-to-training error ratio. The 150×150 configuration is adopted for the subsequent analysis.

*N*	Val. Grid	$d_{\mathrm{norm},\mathrm{NN}}$	${\mathrm{RMSE}}_{\mathrm{tr}}$	RMSE	MAE	MAXE	$\mathrm{RMSE}/{\mathrm{RMSE}}_{\mathrm{tr}}$
			[rad/s]	[rad/s]	[rad/s]	[rad/s]	
100	99×99	$7.07\cdot 10^{-3}$	$5.59\cdot 10^{-5}$	$1.06\cdot 10^{-2}$	$1.36\cdot 10^{-3}$	$1.52\cdot 10^{-1}$	189.7
150	149×149	$4.71\cdot 10^{-3}$	$4.59\cdot 10^{-5}$	$8.00\cdot 10^{-5}$	$4.14\cdot 10^{-5}$	$1.12\cdot 10^{-3}$	1.74
200	199×199	$3.54\cdot 10^{-3}$	$3.90\cdot 10^{-5}$	$4.26\cdot 10^{-5}$	$3.02\cdot 10^{-5}$	$3.81\cdot 10^{-4}$	1.09

**Table 3:** Filtered angular-rate reconstruction metrics under additive white Gaussian measurement noise, with standard deviation equal to 0.5% of the RMS current-readout amplitude, applied to the MEMS current-readout channel of the adopted 150×150 network, for several low-pass cut-off frequencies $f_{c}$. Mean and standard deviation are reported over $N_{\mathrm{MC}}=30$ noise realizations. The first row reports the unfiltered instantaneous estimate. NRMSE statistics exclude runs with |Ω|≤0.5rad/s.

$f_{c}$ [Hz]	RMSE Mean	RMSE Std	NRMSE Mean	NRMSE Std
	[rad/s]	[rad/s]		
no filter	$1.325\cdot 10^{-1}$	–	–	–
500	$5.624\cdot 10^{-2}$	$7.22\cdot 10^{-4}$	2.55%	0.05%
300	$4.213\cdot 10^{-2}$	$6.01\cdot 10^{-4}$	1.89%	0.05%
200	$3.386\cdot 10^{-2}$	$5.56\cdot 10^{-4}$	1.51%	0.04%
150	$2.910\cdot 10^{-2}$	$5.45\cdot 10^{-4}$	1.28%	0.04%
100	$2.367\cdot 10^{-2}$	$5.46\cdot 10^{-4}$	1.03%	0.04%

## Data Availability

The data presented in this study were generated by numerical simulation of the models described in the paper and are available on request from the corresponding author.
